# Matching gerontechnologies to independent-living seniors’ individual needs: development of the GTM tool

**DOI:** 10.1186/s12913-018-3848-5

**Published:** 2019-01-11

**Authors:** Marc Haufe, Sebastiaan Theodorus Michaël Peek, Katrien Ger Luijkx

**Affiliations:** Department of Tranzo, School of Social and Behavioral Sciences, Tilburg University, Tilburg, The Netherlands

**Keywords:** Gerontechnology, Assistive technology, Aging in place, Service delivery, Assessment of healthcare needs, Matching

## Abstract

**Background:**

Most seniors wish to live independently for as long as possible. Gerontechnologies such as personal alarms or remote control systems, have the potential to help them age in place. For seniors, assessing what is the most appropriate technology for their aging in place needs can be difficult. Professionals specifically tasked with matching seniors’ needs with technology solutions can greatly help here. Yet not much is known about the challenges these professionals face or how they can optimize their matchmaking service.

**Methods:**

Participatory action research was conducted in the Netherlands, in two phases. In phase one, ten matchmaking dialogues between municipal technology consultants and seniors were observed, followed by interviews with both technology consultants and seniors to understand the current matchmaking service. In phase two, a new matchmaking tool was co-created with technology consultants and other professionals over the course of four co-creation session. Variants of the tool were tested out in nine additional matchmaking dialogues. The Cycle of Technology Acquirement by Independent-Living Seniors (C-TAILS) model, which can be used to understand both origins and consequences of technology acquirement by independent-living seniors, was used as a theoretical lens.

**Results:**

Important challenges for municipal technology consultants in their current matchmaking practice are: making the matchmaking service more demand oriented and creating an accurate and complete overview of relevant factors within the seniors’ individual situation so that an optimal match can be made. Together with technology consultants and other professionals, a new Gerontechnologies Matchmaking (GTM) tool was created to help overcome these challenges. Evaluation of the tool showed that it better includes each senior’s personal, social, physical and technological context, within the matchmaking service.

**Conclusion:**

Professionals who help seniors match gerontechnology to their aging in place needs experience a variety of challenges in the delivery of their service. Currently, few tools are available for them to overcome these challenges. The newly developed GTM tool can help overcome challenges and optimize matchmaking services. Further testing of the tool in different contexts is needed to determine its generalizability.

**Electronic supplementary material:**

The online version of this article (10.1186/s12913-018-3848-5) contains supplementary material, which is available to authorized users.

## Background

Most seniors want to keep living independently for as long as possible [1–4]. This desire is generally referred to in the literature as *aging in place* [5–8] and has been defined as: ‘remaining living in the community, with some level of independence, rather than in residential care’ [9]. From a policy perspective, stimulating aging in place also makes sense, as aging populations on the one hand and a shortage of care professionals on the other, necessitate finding alternatives to institutionalized care [10, 11]. For these reasons, governmental health and welfare agencies around the world have been encouraged by the World Health Organization to adopt policies to make living places more age friendly [10]. Within this policy framework, the promotion of gerontechnology has become increasingly important [11–15]. Gerontechnologies, sometimes also referred to as assistive technologies, are held to be useful for a host of aging in place purposes, such as the support of daily activities, facilitation of social connection and communication and the enhancement of mobility, personal health, physical and cognitive activity and safety [16–19].

In recent years, a range of gerontechnologies supporting aging in place, such as medication reminders, senior specific mobile phones and tablets, and vital signs monitoring and fall detection systems, have been offered more widely to seniors [19–21]. In addition, ever more businesses, great and small, have started to develop a greater variety of gerontechnology types and variants, as they are increasingly seen as a viable business opportunity [22–24]. This greater variety of gerontechnology propositions could potentially benefit seniors in that they, more and more, will be able to choose a specific gerontechnology or set of gerontechnologies that best fit their needs, wants and living situation. It could also lead to them to not being able to see the forest for the trees however. Recent evidence suggests that seniors prefer the help of an intermediary in trying to find the best match between aging in place needs and gerontechnologies [25].

In the Netherlands, offering of gerontechnology for aging in place purposes is increasingly done by welfare and technology consultants and district nurses. They mostly operate within the legislative framework of the Social Support Act, as one of the main aims of this act is to encourage seniors to live independently longer [26]. Through this policy, care and welfare professionals are expected to make a shift from providing care to supporting seniors to take care of themselves [27]. Professionals tasked with matching seniors’ needs with existing gerontechnologies can play an important role here [28].

Matching seniors’ (implicit) needs with existing gerontechnologies is likely to present professionals with challenges however. It requires not only an understanding of technological offerings that are available, but also of seniors’ needs and circumstances. Seniors are a highly heterogeneous group [29–32]. Research suggests that this heterogeneity is also reflected in seniors’ use of and attitudes towards technology [8, 33, 34]. Seniors vary for example in the degree to which they are technology minded, in their self-efficacy with regards to using gerontechnology, and may or may not view gerontechnology as stigmatizing [35]. By not taking factors like these into account when trying to assist seniors, intermediaries risk discussing gerontechnology in a way that is off-putting, even though that technology could be very suitable for seniors’ aging in place needs [36]. A recent longitudinal field study showed that technologies that are acquired in ways that are not congruent with seniors’ personal needs and circumstances run a higher risk of proving to be ineffective or inappropriate [8]. In order to help ensure that gerontechnologies are effective and appropriate, matchmaking by intermediaries needs to be person-centered and mindful of seniors’ technology specific attitudes and beliefs [30].

Considering the above, we set up a qualitative study to better understand the challenges of intermediaries working to match gerontechnologies to seniors’ aging in place needs, and co-create with them a matchmaking tool to overcome these challenges in their real world practice. Actual choices concerning how best to match gerontechnologies to seniors needs were made while developing and using the tool. This study reports the different steps and findings of our tool development process.

## Methods

### Study design

We adopted a participatory action research (PAR) design with co-creation methodology. Within PAR, researchers and professionals together aim to understand and improve upon a practice by directly engaging in that practice, analyzing and changing it, and evaluating these changes, each form their own perspective [37, 38]. Through co-creation, much exchange between these perspectives is aimed at the construction of new ways of working [39, 40]. In so doing, scientific and practical knowledge are integrated, allowing that knowledge to become more actionable [41].

**Fig. 1:**
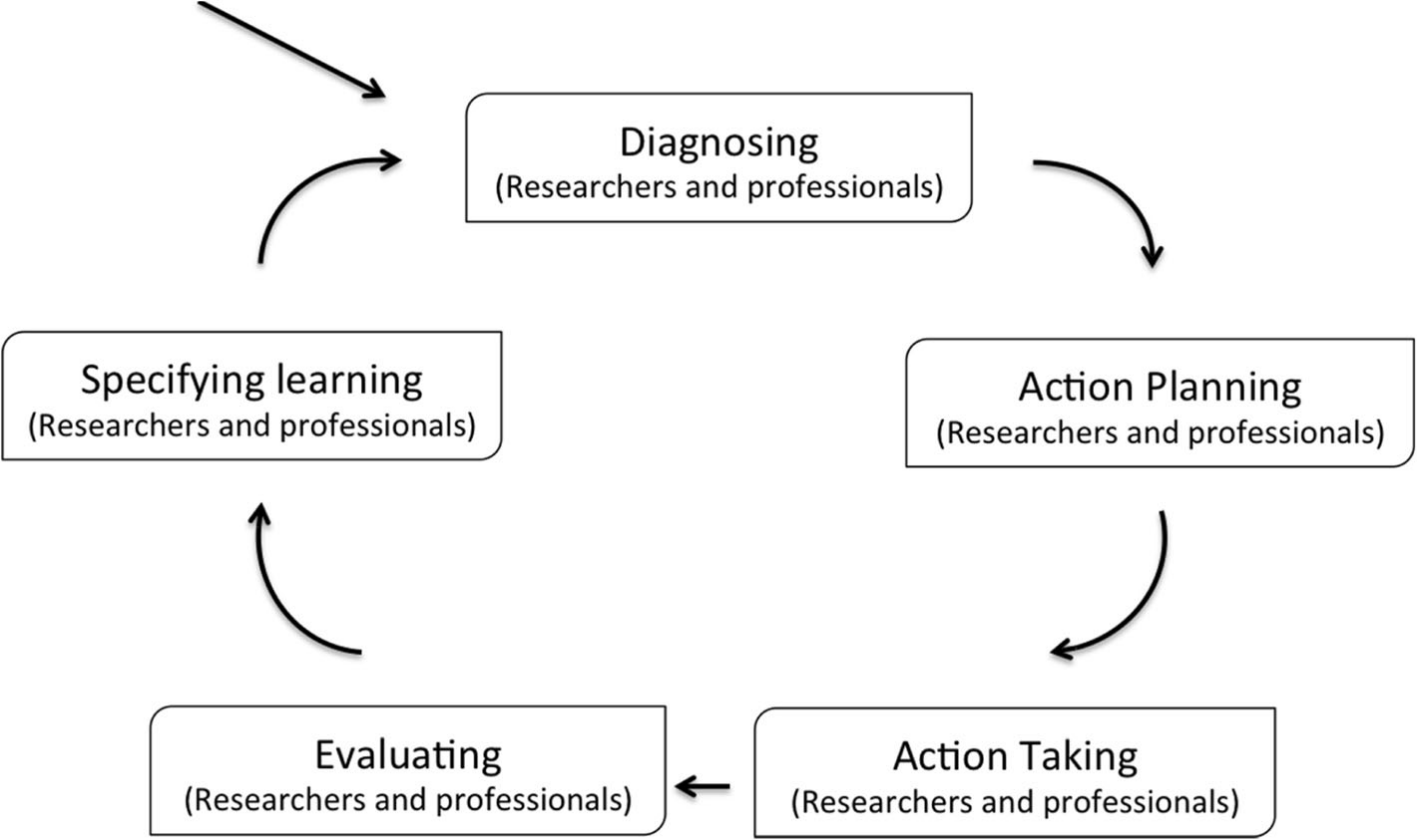
the action research cycle, adapted from [37]

Our study consisted of two phases: (1) understanding the current matchmaking service, and (2) tool development through co-creation. In phase one researchers observed instances of gerontechnology matchmaking involving technology consultants and independently living seniors, and interviewed them separately to gain a better understanding of the current practice. In phase two, aided by a shared understanding of this practice and aided by previous research, researchers and technology consultants worked together in four co-creation sessions to create tool aspects and variants and try them out in practice. Researchers and consultants participated collectively in two action research cycles. Each action research cycle consisted of five stages: diagnosing, action planning, action taking, evaluating, and specifying learning (see Fig. 1) [37].

Prior to engaging in research activities, ethical approval for the study was gained from the ethics review board of Tilburg School of Social and Behavioral Sciences.

### Setting and action research team

The study took place between May 2017 and April 2018 in the municipality of the Hague, the Netherlands. Within the so-called “IZI-Healthy Living at home” pilot project, the municipal government offered a range of gerontechnologies to seniors living independently in five social housing buildings, in order to evaluate them on feasibility, actual use and effects on aging in place. Gerontechnologies ranged from appliances regarding safety (e.g., electronic door locks, in-home sensor systems) to mobility and social interaction solutions (e.g., senior smart phones) to home adaptions (e.g. handrails and grab bars). All gerontechnologies were on view for seniors during guided tours in a model home kitted out with over ninety different items. Seniors were offered to experience technologies in their own home for free for one year. The municipality used this pilot testing to determine which gerontechnologies should be implemented throughout the city. The IZI project had already been in progress a little over a year before the PAR project took place.

In the pilot, technology consultants were tasked with determining if and how specific matches between seniors’ independent living needs and gerontechnologies could be made. Within this setting, researchers and technology consultants, as well as the pilot project manager, worked closely together to develop a tool that could improve the matchmaking service. Additionally, municipal community builders, also tasked with explaining gerontechnology possibilities to seniors within the neighborhood, contributed to the co-creation of the tool. Together, the aforementioned professionals and researchers formed the action research team.

**Fig. 2:**
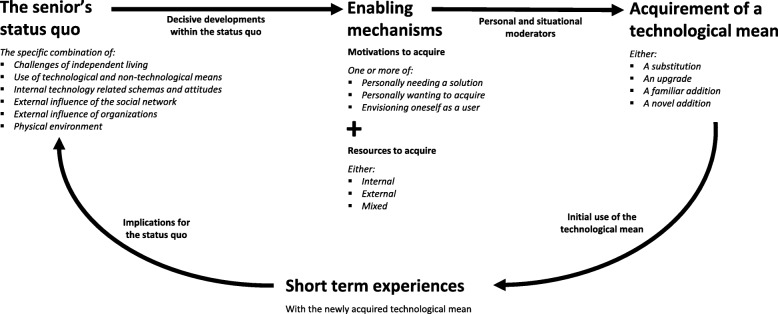
Cycle of Technology Acquirement by Independent-Living Seniors (the C-TAILS model) [8]

Looking at the researchers, they had just finished and published a longitudinal field study on technology acquirement by independent-living seniors. Results had been accumulated in a conceptual model (see Fig. 2): the Cycle of Technology Acquirement by Independent-Living Seniors (C-TAILS) [8]. The C-TAILS model shows the various way in which independent-living seniors’ acquire technologies, and how these lead to successful or unsuccessful use. In the model, acquirements originate from an independent-living senior’s specific status quo and decisive developments within that status quo. These decisive developments can subsequently trigger acquirement enabling mechanisms. Furthermore, acquirements are influenced by personal and situational moderating factors. The longitudinal field study and C-TAILS model inspired the researchers to start conducting research on matching technologies with seniors.

### Sampling

The manager of the IZI pilot project reached out to the researchers because of mutual interest to improve upon the gerontechnology matchmaking service. Technology consultants and community builders were approached by the pilot project manager and researchers and provided with verbal and written information concerning the study. All (two) technology consultants and (three) community builders, agreed to participate. Subsequently, purposive sampling was used to recruit seniors. Seniors needed to live independently at home, be sixty years or older, and be open to discussing gerontechnology. Seniors were asked to participate by community builders and researchers during guided tours in the model home. Interest in the guided tours was stimulated through newsletters, door to door visits by researchers and community builders and community outreach activities of community builders. At the end of the guided tour, seniors were provided with verbal and written information about the study, and were given a week consideration time. All seniors who wished to proceed with a matchmaking dialogue with a technology consultant, also agreed to partake in the study. Seniors were asked to sign a consent form if they wished to participate. Nineteen seniors participated in the study (ten in phase one, nine in phase two).

### Data collection and co-creation

#### Phase one: Understanding the current matchmaking service

In phase one, researchers sought to understand the current matchmaking service through observing matchmaking dialogues and conducting semi-structured interviews with technology consultants and seniors. Observations took place during matchmaking dialogues conducted by the technology consultants with seniors in their homes. Researchers took notes using a preconceived observation guide. Observations focused on understanding the basic structure and dynamic of the matchmaking process. Special attention was paid to how the technology consultants assessed the needs of seniors and how they engaged seniors in trying to ascertain which of the many gerontechnologies on offer would be most suitable in relation to those needs. Semi-structured interviews with ten seniors and two technology consultants were conducted following each of the observed matchmaking dialogues. Topics in interviews with both seniors and technology consultants included: goals and motivations concerning the matchmaking dialogue, satisfaction or dissatisfaction with how the matchmaking dialogue was conducted and views on the outcomes of the matchmaking dialogue (see Additional file 1 for interview guides). Interviews lasted twenty-four minutes on average and were audiotaped and transcribed verbatim. Ten interviews were enough to reach data saturation concerning a basic understanding of the matchmaking service. After the completion of phase one, an analysis was made of the matchmaking service and important issues within that service. This analysis served as input for phase two.

#### Phase two: Tool development through co-creation

Phase two consisted of four co-creation sessions and the trying out of iterations of the tool in nine matchmaking dialogues. These nine matchmaking dialogues amounted to a pilot implementation, in which matchmaking choices by consultants and senior’s were made using the newly developed matchmaking tool. Co-creation sessions lasted on average two hours. Present in all sessions were the two technology consultants, two researchers, the project manager and the community builder involved with the guided tours in the model home. These professionals formed the core of the action research team. Three other community builders attended one or two sessions.

Co-creation session one was conducted to reach a shared focus with regard to the possible/needed improvement of the matchmaking process. Phase one results were used to create a shared understanding of the current process. Additionally, the session served as a team building exercise. Researchers first involved participants via a creative method to make the experience or “embodied knowledge” of participants more explicit. Embodied knowledge concerns routines, habits tasks and information, which are understood without much conscious thought [42]. Participants were asked prior to the session to select two images or objects that to them symbolized the current and their ideal matchmaking process. At the beginning of the session, each participant presented their selection, explaining their rationale for choosing these images or objects. Participants reflected on what they saw and heard, and what the group saw as the main characteristics of the current and ideal matchmaking service, as well as the main differences between the two was noted on a flip-over chart. Next, the researchers shared results from phase one to further explore elements of the matchmaking service. Additionally, a researcher presented the C-TAILS model (see Fig. 2). The session ended with an exploration of ways in which this knowledge could be used to realize the group’s ambitions and overcome challenges.

Co-creation session two was held a week later to further develop ideas to improve the matchmaking service and make these ideas applicable in practice. One researcher presented the most important ambitions and first ideas that had emerged in session one in a logical order. Members recognized themselves strongly in this narrative and added a few new ideas for possible improvement. In the ensuing discussion, consensus was reached with regard to which ideas in where deemed most promising to include in a matchmaking tool. In the second part of the session, two further exercises where carried out to facilitate the co-creation of the matchmaking tool. First, a researcher presented more in depth insights from the C-TAILS model. In exploring more thoroughly personal and contextual factors that may impact successful technology acquirement and use by seniors, informed decisions could be made about topics that should be included in matchmaking tool. Second, the group created a “client journey” with all moments of contact between seniors and members of the IZI project team leading up to and including the matchmaking dialogues with technology consultants [43]. As a result of these two exercises, the group was able to determine who knew what and when about seniors’ individual needs and circumstances. This also led to a discussion about how working arrangements between project members should be optimized to accommodate the implementation of the matchmaking tool. Based on these insights, the basic structure and content of the tool, as well as new ways of working together, were established.

Following co-creation session two, a first draft of the tool was created and shared within the action research team, enabling all members to provide feedback and suggest changes. In between co-creation sessions two and three and three and four, two iterations of the matchmaking tool were tested in their real world practice by one community builder and two technology consultants. They were asked to make notes about their experiences in using the tool, in particular, ease of use and perceived effectiveness within the matchmaking service.

Co-creation sessions three and four were conducted to further fine tune and evaluate the tool, focusing on its acceptability, appropriateness and feasibility in practice [44]. The structure of these sessions was similar. First, the community builder and technology consultants shared their experiences with the tool so that aspects could be improved upon. Herein the focus was on increasing the usability and shared understanding of the tool. Second, the tool was evaluated by relating its use to the goals for improvement agreed upon in co-creation session one. Apart from the matchmaking tool, the process of co-creating together PAR was also evaluated. Researchers took care to ensure that all group discussions took place within constructive group dynamics. All questions asked were from a position of appreciative inquiry, non judgmental and in affirmation of the strength and potentials of the action research team members [45]. This allowed the co-creation sessions to become “safe spaces” [37], where all members felt safe enough to speak freely and openly with one another, sometimes even with (heavy) criticisms of the current process.

### Data analysis

Thematic analysis was used for the data collected in phase one, using qualitative data analysis software (Atlas.ti version 8) [46]. First inductive codes were attached to quotes relevant to the research questions. Transcripts were coded independently by two researchers, with one researcher coding twelve of the twenty transcripts, and another researcher eight. Second, researchers came together to discuss their codes in the form of case studies and codetermined overarching themes. Phase two results, were continuously analyzed within the action research group in each co-creation session. The three most important steps for analysis were: codetermining the collective goals for improving the matchmaking service, codetermining the specific characteristics of the matchmaking tool and co-evaluating the use of the tool.

## Results

### Sample

Concerning the seniors, the sample consisted of nineteen participants who were aged in their sixties, seventies and eighties (average age: seventy four). The sample contained more women (eleven) than men (eight), and the majority (fifteen) lived alone. Of the four seniors who lived together with their partners, all partners where present during the matchmaking dialogues. For two seniors living alone a daughter was present during the matchmaking dialogue. Most participants had enjoyed lower education only. Three participants were not fluent in Dutch and required the presence of an interpreter to help them. Members of the action research team were aged in their thirties, forties and fifties (average age: forty six). All were highly educated and experienced in their respective fields.

### Phase one: Understanding the current matchmaking service

Through the observations of matchmaking dialogues and subsequent in-depth interviews with both seniors and consultants, three issues in particular were found significant: the tension between a supply and demand driven approach, the role of informal caregivers and the timing of the matchmaking dialogue.

#### Tension between a supply and demand driven approach

In trying to ascertain which gerontechnologies where most beneficial to seniors in facilitating aging in place, the IZI project sought to test a wide array of technologies. At the same time a quota was established for how many units of a particular gerontechnology should be tested within the confines of the project. Because of this, it was found that technology consultants regularly sought to match gerontechnologies that had not yet been distributed widely, even if the seniors’ need for them was not high.

*‘… There is a tension between: do people need something and how many technologies do we want to test in this project. There are a few things we simply want to try out with people, so we give it to them, even though they might not really need it.’* (technology consultant)

Additionally, because certain gerontechnologies had already been matched frequently in the first months of the IZI project, technology consultant were not able to offer them to seniors any longer.

*‘…You can’t always match up their needs with what we want, I know that, for instance they wanted those mobility aids, but they aren’t distributed anymore. That’s a shame really.’* (technology consultant)

The aforementioned regularly impeded the matching of technologies with seniors most important needs and wants.

#### Role of informal caregivers

Apparent within the observed dialogues was also the role of significant others, such as partners or children, who acted as caregivers for the senior. Caregivers influenced matchmaking mainly in two ways. First, they often helped the senior with trying to find the right gerontechnology solutions.

*‘...I just really liked to be there for her, to support her you might say, but also to give my perspective on things.’* (caregiver)

Second, they also regularly had specific needs themselves that could also be met by a certain gerontechnology.

*‘… like [the electronic locks on] the door, it is important for us to be able to gain access quickly to see if she is all right.’* (caregiver)

Instead of trying to find a match between one person’s needs and gerontechnology, technology consultants in these cases tried to accommodate two sets of needs, sometimes complicating the matchmaking service.

#### Timing of the matchmaking dialogue

Lastly, for a few seniors, the matchmaking dialogue was not conducted at an appropriate time for them, even though they had agreed to participate. One senior felt overwhelmed by recent life events (such as a move to a new home and health problems), and therefore repeatedly indicated that she was not up to the task of determining whether gerontechnology might be helpful to her.

*‘…I don’t know yet, as I said, I am too preoccupied with the move. I don’t feel like myself… I have to go to three medical appointments; to the orthopedist, to the lung doctor and I have just been to the cardiologist.’* (senior)

For other seniors, the matchmaking dialogue was optimally timed. For example, some seniors had recently experienced challenges to their independence and thus were highly motivated to find a gerontechnology solution. In these ways timing could greatly impede or facilitate the matchmaking service.

### Phase two: Tool development through co-creation

In phase two, the action research group (researchers, technology consultants, community builders and the IZI project manager) ran through all stages of the action research cycle: diagnosing, action planning, action taking, evaluating, and specifying learning (see Fig. 1). Findings in each stage are described in detail below.

#### Diagnosing

**Fig. 3:**
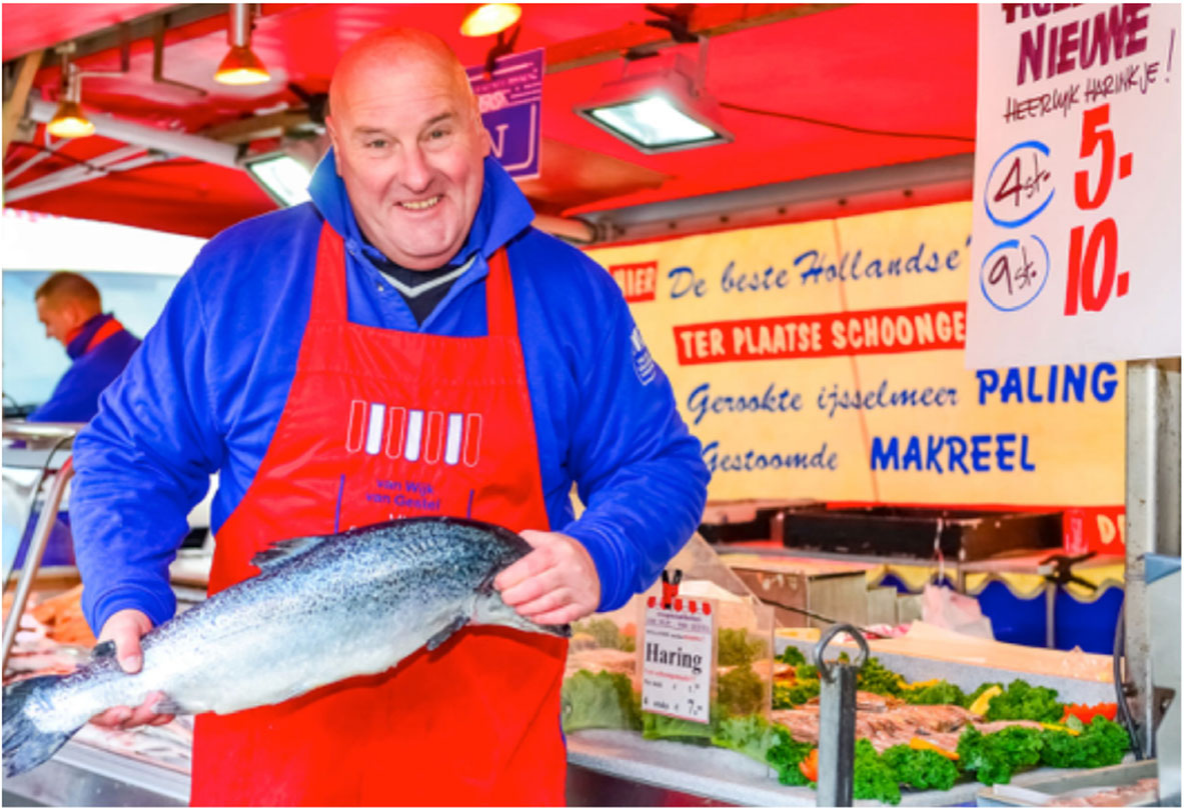
Image symbolizing the current matchmaking process, photo courtesy of Jeroen Vos [stock photo] [47]

By discussing imagery selected by team members symbolizing the current and ideal matchmaking service, five areas of improvement could be diagnosed. First, multiple people in the team felt that the current process was too supply driven, focusing on “selling” the technology, and less on meeting individual needs. This was seen as symbolized by an image of a market vendor that advertises his wares (Fig. 3). An important improvement for the group therefore was to make matchmaking more demand oriented.

**Fig. 4:**
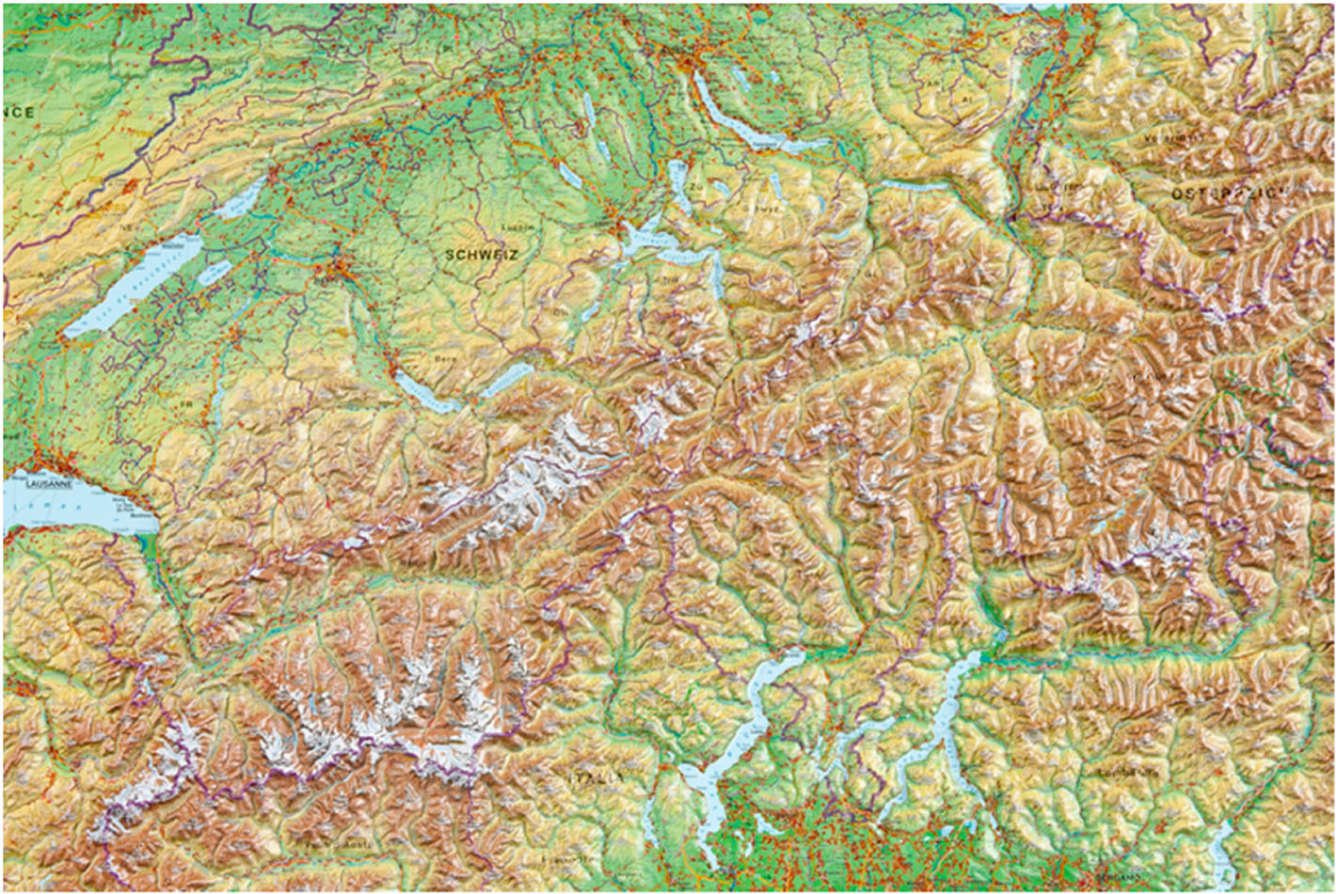
Image symbolizing the ideal matchmaking process, photo courtesy of Georelief [48]

Second, the design of the current matchmaking service was seen to be geared towards efficiency, symbolized by the group members’ image of a car navigational system, an instrument to help reach a destination as quickly as possible. Through such navigation however, the current service was said to induce tunnel vision: by focusing on needs that could be matched quickly with a gerontechnology on offer, technology consultants did not strive to fully understand the situational context of seniors’ needs. One member saw the ideal matchmaking service as a map, conducted with a “helicopter view” of all factors that might impact a senior’s need for and use of gerontechnologies (Fig. 4). Another important improvement for the developmental group was therefore to achieve more of an overview of a senior’s situation.

Third, it was acknowledged that the role of informal caregivers within the current matchmaking service should be understood better and should therefore be part of the overview. Fourth, the current service was symbolized by a member as being populated by a lot of well-meaning professionals who did not always communicate well together regarding their contacts with seniors. Because of this, the matchmaking service sometimes ran less smoothly, as follow ups to initial contacts with seniors did not take into account what had been discussed with seniors earlier. A point of improvement was therefore more efficient communication between all professionals working to achieve a match between a gerontechnology and a specific senior. Fifth, the issue of timing. Implicit within the current matchmaking service seemed to be the assumption that seniors’ needs and circumstances were fairly static, not changeable over time. Because of this, only one matchmaking dialogue per senior was conducted, and seniors were not monitored as to whether their needs or situation changed. Ideally, there would be continuous contact between professionals and seniors, so that the team can determine when the timing is best to conduct a matchmaking dialogue. This too was seen as an improvement point.

Through subsequent presentations of the researchers of phase one results and the C-TAILS model, the above improvement points where fleshed out. The group subsequently decided as goals for the matchmaking tool: (1) achieving a better balance between supply and demand, by (2) matching at the right time, (3) with a better overview of a senior’s situation, (4) whereby the informal caregiver is given greater attention, and (5) team members communicate more efficiently with each other.

#### Action planning and action taking

The action research team discussed ways in which the goals could best be reached. Two related ideas in particular were judged to be the most promising and workable for a matchmaking tool: (1) developing a new matchmaking dialogue guide to achieve a more complete overview of the current situation of a senior and their primary caregiver, and (2) developing a way of working together whereby more team members contribute to and share this overview. Concerning the first idea, more in-depth sharing of aspects of the C-TAILS model allowed the group to judge which additional factors should be included in the matchmaking dialogue guide. Concerning the second idea, a client journey was created and reflected upon. It showed that the community builder frequently became aware of recent life events or attitudes towards gerontechnology when conducting guided tours in the model home, but that he did not share these insights. Technology consultants stated that this prior knowledge could help them in their preparation for the matchmaking dialogue. In reflecting, the team also spontaneously formulated ideas about what types of questions should be asked, by whom, in order to attain a better overview of a senior’s situation. As such, the new tool added a range of new contextual and gerontechnology specific questions to the old way of working. Foremost among these were: recent developments in seniors lives, general attitudes towards gerontechnology, the level of satisfaction with current means used to fulfill needs in various domains, seniors ideas about the properties of match worthy gerontechnology and the expected consequences of the use of that gerontechnology. In addition, the original task of working through a list of all available gerontechnologies with seniors was taken out. Also, the new guide was restructured so that both community builder and consultants could use it in their contacts with seniors. Lastly, all questions were made to pertain both to the senior and, if applicable, their primary informal caregiver.

#### Evaluating

**Fig. 5:**
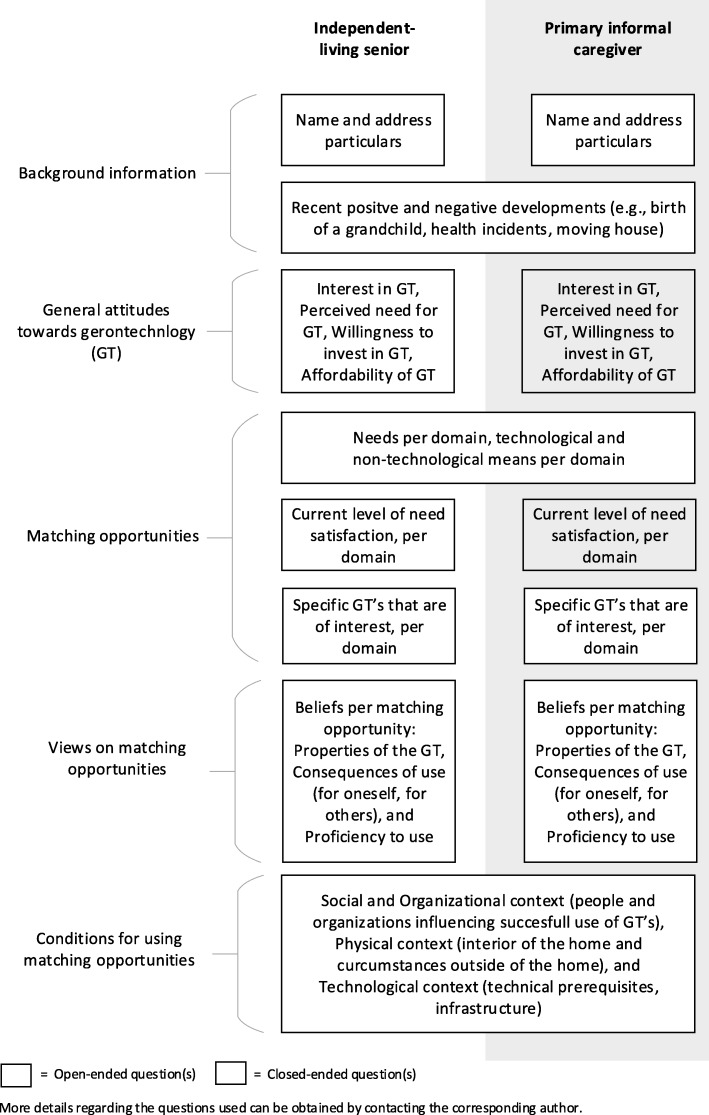
Final outline of the GTM tool

With regard to usability in real world practice, the first iteration of the matching tool took some time getting used to. For the community builder it was not always clear which parts of the tool he could use. It was decided that during the second iteration he should focus on recent developments and attitude towards gerontechnology because the guided tour setting was best suited to assess these two factors. For technology consultants, strictly following the order of the new tool felt a bit unnatural. It was therefore agreed upon during the second iteration that consultants had a certain leeway in the specific formulation and order of questions during their contact with seniors, as long as the obtained answers gave a clear and accurate picture concerning all factors included. In this way, consultants could maintain their personal approach to the dialogue. However, a fixed order for the factors: views on matching opportunities and conditions for using matching opportunities was maintained during the second iteration of the tool. To increase a shared understanding of the guide, some segments were also renamed, and examples of certain terms were added to the guide to make their meaning more explicit. Lastly, an agreement was reached about which specific domains of independent living should be assessed during the matching service. The testing of this second iteration in real world practice yielded satisfactory results and no further changes were made for the final version of the tool. The final outline of the resulting Gerontechnologies Matchmaking (GTM) tool is presented in Fig. 5.

In comparison to the old way of working, the new way was seen as providing a better balance between a supply and demand driven approach. The new tool focused more on the specific needs of seniors and caregivers and less on all technologies on offer:

*‘… in the old situation I used to run through my list with technologies asking things like: what do you think of that? or maybe this [technology] is something for you? That is something I didn’t do anymore’* (technology consultant).

Use of the new tool was also found to improve obtaining a more complete overview of a seniors’ situation:

*‘… now you also look at how vulnerable the situation is, how many problems there are potentially [with a certain gerontechnology match]* (technology consultant)

Additionally the more systematic inclusion of the role of informal caregivers was noted:

*‘…It is part of the registration stream, that’s something that we didn’t do previously.. now we always explicitly check that [the role and influence of the caregiver].’* (project manager)

The community builder saw that such a stream could potentially help him with follow up questions concerning the installed gerontechnology matches. Consultants felt that a more systematic way of involving informal caregivers was introduced, yet also felt they had already started to engage the caregivers more, prior to the use of the new tool.

Furthermore, communication between the community builder and the consultants had been improved by the shared use of the new tool. Technology consultants liked the fact that they received more information prior to home visits:

*‘…You can build on pre-information that you get from the guided tours. It can be advantageous to have that*’ (technology consultant)

However, consultants did feel however that they should not receive too much pre-information, in order to keep an open mind when going into the matchmaking process.

With regard to the improving appropriate timing of matchmaking dialogues, no changes were made in comparison to the old way of working. All interested seniors continued to be directly referred to technology consultants, because a senior’s expressed interest in technology continued to be seen by most team members as a signal that the timing was right to offer gerontechnology. A few team members did acknowledge that a more continuous monitoring of a seniors’ situation could ensure better timing of the matchmaking dialogue. However, they also concluded that such monitoring was not feasible due to the limited time available for community builders.

#### Specifying learning

In reviewing the outcomes of the evaluating stage, the action research team was able to specify how the new tool could be applied more broadly. Two variants were discussed: the application of the tool by the same team in a different area of the municipality and the application of the tool by professionals other than the action research team.

The guide was deemed directly transferable to a new area with the same team, although differences in project arrangements might necessitate a slightly different approach. The practice of providing guided tours for seniors of a model home before conducting matchmaking dialogues for instance, was not viewed by all to be an essential step in a new area. Concerning the application of the tool by other professionals, it was concluded by most that a short training should be provided. An oral explanation of the tool, a review of one or more case studies, and a practice round were recommended. The team was divided as to the level of experience and technical expertise necessary to successfully work with the tool.

## Discussion

In this study a first version of a tool that may enable gerontechnology intermediaries (e.g., such as welfare and technology consultants and district nurses), to better match gerontechnologies to senior’s individual aging in place needs and circumstances was developed. The GTM tool can be used to create a relevant overview of factors such as recent life developments, gerontechnology- related attitudes and beliefs, and the level of satisfaction with current solutions. As such, this overview can help intermediaries determine whether gerontechnology is suitable, which gerontechnology or set of gerontechnologies best fits the situation of a senior, and what type of support may be needed for gerontechnology use. This is significant, because there is an absence of matching tools for these purposes in the aging in place literature [30, 49].

This type of tool is not entirely without precedent however. Within literature concerning the rehabilitation practices for people with disabilities, one matching tool, or rather set of tools, is mentioned regularly: the Matching Person and Technology (MPT) Assessment Process [50, 51]. The underlying Matching Person and Technology model emerged from grounded theory research that sought to define which areas should be assessed to allow for an adequate match between assistive technologies (e.g., wheelchairs, adapted utensils, communication devices) used to increase or maintain functional capabilities, and the needs and preferences of a person with a disability. Several assessment tools were subsequently developed by Sherer and colleagues for determining the disabled person’s needs and preferences, environmental factors influencing use, and functions and features of the most desirable and appropriate technology [52]. The tools take the form of a pen and pencil measure that can also be used as a dialogue guide.

The MPT tools are similar to the GTM tool in that both seek to understand more about a prospective users’ attitudes and prior experiences of technology in trying to find the right match. The GTM tool differs from the tools within the MPT assessment process in that it is specifically designed for independent-living older adults, with and without disabilities.

In addition, the GTM tool structurally involves informal caregivers in the matching process by also assessing their experiences and attitudes, because of the noted importance of these caregivers in senior’s lives. Finally, the GTM tool pays attention to seniors’ and informal caregivers’ willingness to invest in the acquirement of relevant technologies. This feature is deemed especially important in light of the ongoing governmental effort in the Netherlands and other countries to stimulate seniors’ independence in a cost effective way.

There are a number of limitations that must be considered with regard to this study. Due to time constraints, a limited number of intermediaries and seniors were recruited through our partnership with the IZI pilot project. Co-creation thereby focused on the matching service of technology consultants, as other type of intermediaries’ such as district nurses were not involved with the pilot project. Also, participating seniors resided in social housing flats and tended to have enjoyed lower education only. More research is needed to determine to what extend the GTM tool is acceptable, appropriate and feasible in other contexts. Lastly, the GTM tool can be validated in a randomized controlled trail to test its effect on seniors’ acceptance and use of gerontechnologies as well as health-related outcomes.

There are also a number of strengths of this study. Particularly in the last decade, co-creation has become increasingly popular in several fields, including business studies, design science, computer science, and community development [39]. However, co-creation remains underutilized when it comes to research in the field of gerontechnology [53]. The current study is the first that we know of that sought to specifically unearth challenges of intermediaries in matching gerontechnologies to seniors’ independent living needs and circumstances. By utilizing PAR and co-creation principles a tool was developed that explicitly incorporates the knowledge and experience of technology consultants, thereby making it more tailored to the real world practice of such intermediaries. The use of a creative method to involve intermediaries in the analysis of their practice was particularly helpful in this regard. Furthermore, by drawing on the insights of the C-TAILS model, the action research team was able to incorporate a host of contextual factors concerning the acquirement and use of gerontechnologies by seniors into the tool. Finally, the testing of different variants of the tool in iterations of the action research cycle, allowed for a more in-depth evaluation of its usability and effectiveness in light of the stated improvement goals.

## Conclusion

Our co-created GTM tool allows the matching of gerontechnologies to seniors aging in place needs to be more person centered, to achieve more of a comprehensive overview of factors that impact gerontechnology attractiveness and use, and to be more inclusive with regard to the role of informal caregivers. Further testing needs to occur with a wider range of intermediaries’ and seniors, to determine the generalizability of the tool as well as its added value for allowing seniors to age in place with the support of technology.

## Additional file

Additional file 1: Phase 1 Interview guides.docx, Interview guides for phase 1, Contains the interview guides for both the municipal technology consultants and seniors. (DOCX 15 kb)

## Abbreviations

C-TAILS: Cycle of Technology Acquirement by Independent-Living Seniors
MPT: Matching Person and Technology
PAR: Participatory Action Research
