# Supplementary material for: Matching gerontechnologies to independent-living seniors’ individual needs: development of the GTM tool
Source: BMC Health Serv Res. 2019 Jan 11;19:26. doi: 10.1186/s12913-018-3848-5 (PMC6329159; doi:10.1186/s12913-018-3848-5)
Supplement: Supplementary file 1 — Phase 1 Interview guides.docx, Interview guides for phase 1, Contains the interview guides for both the municipal technology consultants and seniors. (DOCX 15 kb) [file 12913_2018_3848_MOESM1_ESM.docx]

Phase 1 interview guides

Interview guide municipal technology consultants

- How did you experience the matchmaking dialogue?
- What information about the senior did you have before the start of the matchmaking dialogue?
- What was the goal of the dialogue?
  - How did you want to accomplish that goal?
- What went well, why?
- What did not go well, why?
- Did you encounter any obstacles or resistance?
  - How did you deal with that?
- What was the main need or goal of the senior?
- What were the main results of the dialogue?
  - How do you know?
- What is the follow up within the iZi project?
  - Does it involve colleges, technology suppliers, social services or the senior’s social network, how?
- Will you monitor the situation after the technology is installed?
- Are factors such as experience of use, level of fit to lifestyle, level of satisfaction with technology and / or outcome considered?

Interview guide seniors

- What was the reason for having this conversation with the technology consultant?
- What was the goal of the conversation?
- How did you experience the conversation?
  - What went well, why?
  - What did not go well, why?
  - Which part of the conversation was most useful for you?
  - Which part of the conversation the least useful?
- How do you now view all the technological possibilities in support of independent living?
  - What disadvantages do you see?
  - What advantages?
  - Also check for perceived advantages / disadvantages with regard to: significant others, (agreements with) care and/ or welfare agencies, in-home use, neighborhood use and effort of use
- Does this view of technological possibilities in support of independent living differ from your view prior to the conversation?
  - If so, how?
- What is your most important independent living need?
- How well did the discussed technological options fit this need?
- Do you have a (very) good feeling concerning one or more of the options?
  - If so, why?
- What do you expect from the use of that technology/ those technologies?
